# Should We Stop Calling Thanatophoric Dysplasia a Lethal Condition? A Case Report of a Long-Term Survivor

**DOI:** 10.1089/pmr.2020.0016

**Published:** 2020-05-14

**Authors:** Ricki S. Carroll, Angela L. Duker, Andrea J. Schelhaas, Mary Ellen Little, Elissa G. Miller, Michael B. Bober

**Affiliations:** ^1^ Division of Orthogenetics, Nemours/Alfred I. duPont Hospital for Children, Wilmington, Delaware, USA.; ^2^ Division of Palliative Medicine, Nemours/Alfred I. duPont Hospital for Children, Wilmington, Delaware, USA.; ^3^ Sidney Kimmel Medical College, Thomas Jefferson University, Philadelphia, Pennsylvania, USA.

**Keywords:** lethal condition, pediatric palliative care, perinatal palliative care, prognosis, rare disease, skeletal dysplasia, thanatophoric dysplasia

## Abstract

Thanatophoric dysplasia (TD) is a rare skeletal dysplasia commonly thought to be lethal. In this case report, we discuss a nine-year-old male with TD and review his parents' decision making shortly after their son was born, the technology needed to sustain him, and his parents' perception of his quality of life. We also summarize the clinical course of published long-term survivors with TD. Pediatric Palliative Care teams, especially those conducting perinatal palliative care consultations, are often asked to support families in the face of prognostic uncertainty. Our case report and review of the literature adds to the uncertainty of prognosis in TD and suggests that pediatric palliative care providers should be wary of the label “lethal.”

## Introduction

Thanatophoric dysplasia (TD) is a skeletal dysplasia that was previously described to be incompatible with life. The term *thanatophoric* is Greek for “death bearing.” Infants with this condition have extreme short stature, micromelia, a narrow chest, underdeveloped lungs, macrocephaly, and a small foramen magnum. Occurring in 1/20,000 to 1/50,000 births, it was understood to be lethal soon after birth due to difficulty with ventilation and development of respiratory failure.^1–4^ There are very few reports of patients who have survived beyond the first few days of life; therefore, families faced with this diagnosis are typically counseled to consider pregnancy termination and/or comfort care at birth. However, with advances in technology, survival can be possible for some. This makes counseling around prognosis and expected clinical course challenging for families faced with a perinatal diagnosis of TD.

In this brief report, we describe the case of a boy with thanatophoric dysplasia type 1 (TD1) and review the medical decisions the family faced. We also summarize the clinical course of published long-term survivors with TD. Both serve not only as prognostic guides for this specific condition but also to illustrate themes that emerge when providing palliative care for children with diseases commonly labeled as “lethal.” The family gave permission for their child's name and story to be shared.

## Case Description

Charlie was born to a 33-year-old gravida 7 para 3 mother and a 33-year-old father. Pregnancy was complicated by polyhydramnios and features suggestive of TD by anatomical ultrasound. Amniocentesis identified a c.742C>T (p.R248C) mutation in *FGFR3*, consistent with a diagnosis of TD1. Charlie's mother recalls being given the diagnosis by a high-risk obstetrician and a genetic counselor who both counseled that, if she were to carry to term, the infant would not make it out of the delivery room alive and, therefore, offered termination of the pregnancy. After connecting with two families whose children were long-term survivors with TD, they made the decision to continue the pregnancy. Charlie's mother met with her local neonatology team and together they created a birth plan that supported her goal of prolonging Charlie's life.

Delivery occurred at 36 weeks gestation through repeat cesarean section, and the infant required intubation and ventilator support immediately after birth. He spent five months in the neonatal intensive care unit where he received frequent speech, physical, and occupational therapy. Tracheostomy and gastric tube were placed at two weeks of age. He was treated for subclinical seizures and had challenges with hypoventilation and apnea, all of which improved with time. He was discharged to a rehabilitation facility for one month before going home.

He presented to our skeletal dysplasia program for multidisciplinary evaluation at 2.5 years of age. At that time, he had been generally healthy with one hospital admission for treatment of pneumonia since birth. He was able to roll from his back to his stomach and also used a power wheelchair for mobility. He was able to feed primarily by mouth with assistance, utilizing the gastric tube infrequently. Polysomnography obtained around this time demonstrated periodic breathing and central apnea with oxygen desaturations, and his neurological examination was notable for hypotonia, weakness, hyperreflexia, and crossed adductor reflexes, raising the concern for critical foramen magnum stenosis. Magnetic resonance imaging of the cervical spine and craniocervical junction identified a narrowed foramen magnum and diffuse narrowing of the cervical canal with signal changes within the spinal cord, and he subsequently underwent foramen magnum decompression the following month. After surgery, his developmental skills advanced significantly and his central apneas resolved.

At four years, his gastric tube had been removed and his ventilator settings were being slowly weaned over time. Acanthosis nigricans, a known skin finding in children with changes in the *FGFR3* gene unrelated to glucose intolerance, was first observed in Charlie at approximately two years of age and had begun to spread within skin folds and areas of friction. He would go on to have surgical excision of these skin folds multiple times in an effort to reduce the complications that arose in these areas.

**FIG. 1.**
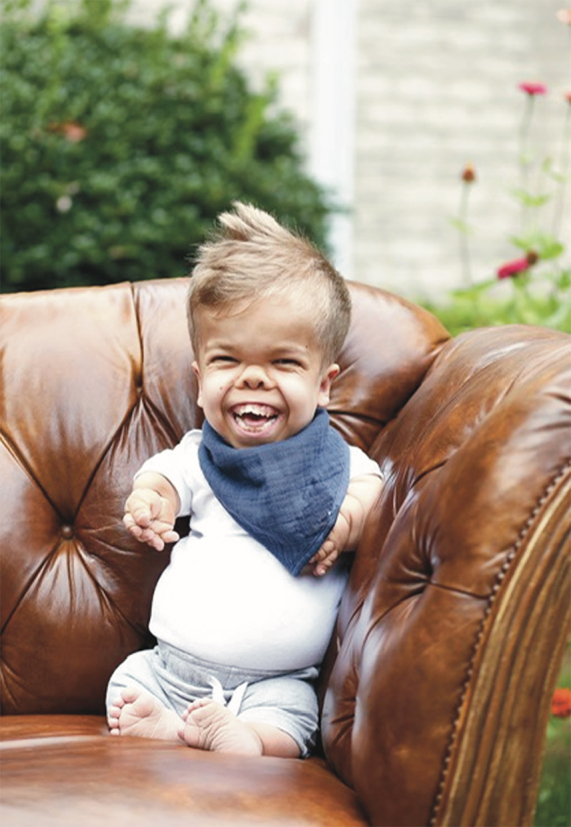
Charlie at nine years of age in the front yard of his home.

Repeat evaluation each year since has demonstrated consistent progression of developmental skills. At seven years of age, he was able to army crawl, bear weight in a jumper, and sit unsupported for one hour. With the assistance of a communication device, as well as sign language, he was able to use up to three-word phrases to communicate his needs and answer questions, including asking for specific foods and activities. Neuropsychiatric testing placed him at the one- to three-year-old level, with the caveat that standardized testing is made for children who experience the world much different than Charlie; therefore, it was noted to better describe him functioning within the sensorimotor developmental stage. Today, Charlie's mother described him as “a thriving 9-year-old boy” (Fig. 1) who feeds himself, drives his own power chair, throws a ball, colors, and paints. He has been weaned off the ventilator since eight years of age, and is now working toward decannulation, of which we believe he would be the first child with TD1 to accomplish.

## Discussion

### Expected clinical course

A crucial role that palliative care providers serve is to help families gain a better understanding of their child's prognosis and expected clinical course. This can be challenging when a fetus or a child carries a rare diagnosis and/or has surpassed prognostic expectations. A summary of the medical needs of published long-term survivors with TD can be found in Table 1.^5–14^ Although the number of published individuals living with TD1 are few, knowing the medical decisions and interventions these children required can be helpful when families turn to us for guidance.^15^

**Table 1.** The Clinical Course and Medical Interventions of Published Long-Term Survivors with Thanatophoric Dysplasia

	Baker et al.^5^	Katsumata et al.^6^ and Kuno et al.^7^	MacDonald et al.^8^ (patient 1)	MacDonald et al.^8^ (patient 2) and Nikkel et al.^9^	Pokharel et al.^10^ (case K); Nakai et al.^11^	Okajima et al.^12^	Stensvold et al.^13^	Tonoki^14^	Charlie
General									
Gender	M	M	M	F	F	M	F	M	M
Age at the time of publication	9 years	8 years	4.75 years	4 years; 28 years	23 years	6 years	169 days	212 days	9.5 years
Current age	Unknown	Unknown	Deceased at 5.2 years	Unknown	Unknown	Deceased at 6 years	Deceased at 169 days	Deceased at 212 day	9.5 years
Mutation in FGFR3	R248C	G370C	Unknown	R248C	R248C	S249C	Unknown	Unknown	R248C
Thoracic circumference	NR	26.8 cm at birth; 35.8 cm at 8 years	23.5 cm at birth; 39 cm at age 3 years 9 months; 42 cm at 4.75 years	24 cm at birth; 41.5 cm at 3 years 9 months	NR	NR	NR	NR	40.8 cm at 4 years 1 month
Head circumference	39.5 cm at birth (+1 SD^a^); −1.7 SD^a^ at 8.7 years	37.0 cm at birth; 46.1 cm at 8 years	33.5 cm at birth; 47.5 cm at 4.75 years	35 cm at birth	NR	35 cm at birth	38.5 cm at birth	NR	60.8 cm at 8.5 years
Linear growth	−6 to −6.5 SD^a^	Length did not increase beyond 49.0 cm after age 5	Absent linear growth after ∼10 months	Absent linear growth after ∼10 months	NR	Almost no linear growth	NR	NR	71.4 cm at 8.5 years
Neurological									
CNS abnormalities	Stable mild ventriculomegaly since 9 months; high cervical myelopathy; hyperreflexia, sustained ankle clonus and upgoing Babinski sign	Mild brain atrophy at age 7	Abnormal gray/white matter differentiation; communicating hydrocephalus; VP shunt placed	Progressive hydrocephalus; VP shunt placed	Moderate ventriculomegaly	Mild nonprogressive ventricular dilation	Progressive hydrocephalus; absent reflexes	Mild cerebral atrophy	Moderate ventriculomegaly; hypotonia, crossed adductor reflexes, no clonus
Craniocervical junction abnormalities	Narrow transverse diameter of foramen magnum and marked upper cervical stenosis	NR	Auditory evoked potentials concerning for brain stem compression at foramen magnum	Progressive stenosis resulting in quadriplegia; MRI at 18 yo demonstrated no CSF flow through the FM	NR	NR	NR	NR	Stenosis of the foramen magnum with cervical cord compression
Decompression surgery	No evidence of cervical cord compression	NR	Yes, no apparent benefit	Yes, in infancy with transient improvement in ventilator need; no further decompression attempted	NR	No	No	No	Yes, postoperative improvement in development and central apneic events
Seizures	Yes, since 7 months of age, controlled with AEDs	NR	Yes, generalized seizure at 3 months	Febrile seizure in infancy, seizure disorder at age 15 controlled with AED	NR	NR	NR	Frequent apneic attacks with cyanosis treated with AEDs	Yes, subclinical seizures in neonatal period
Hearing	Mild to moderate hearing loss, secondary to chronic otitis media; bilateral hearing aid use	NR	Dysfunctional retrocochlear pathway of left acoustic nerve	Right cholesteatoma; significantly impaired hearing; ability to recognize voices and react to sounds	NR	Severe sensorineural hearing loss	NR	NR	Chronic otitis media; bilateral cholesteatoma necessitating multiple surgeries; moderate hearing loss
Respiratory									
Tracheostomy	Yes	No, per family request	Yes	Yes	NR	NR	No	No	Yes
Ventilator dependence	Yes, able to tolerate brief 10- to 12-minute windows with supplemental oxygen	Long-term intubation and mechanical ventilated since birth	Yes	Yes beginning at 4 months; from 8 to 10 years tolerated up to 8-hour window most days; tolerated 30-minute intervals until age 15, when she became fully dependent	Yes	Yes	No, oxygen supplementation	Oxygen support in infancy; periods of apnea noted; ventilator use during illness	Yes previously; however, no longer dependent (since age 8)
Development									
Motor skills	Approximately 2 to 12 months at chronological age of 4.8 years (rolls, scoots, sits unsupported, and pulls to stand with assistance); uses manual wheelchair for mobility	NR	“Profound” developmental delay	Previously able to roll and move around on her abdomen; ∼8- to 18-month level during teen years	NR	Development reported as similar to that described by Baker et al.	NR	NR	Sensorimotor developmental stage (army crawls, sits unsupported for 1 hour, and throws a ball); uses power wheelchair for mobility
Cognitive and language skills	Approximately 18 months at chronological age of 8 years; vocalizations but no distinct words at age 9	Ability to make emotional expressions; language perception estimated at 10 to 12 months at chronological age of 8 years	Approximately 2 weeks at chronological age of 4.75 years; responded to visual cues only	Some vocalizations; ability to express likes/dislikes; use of limited sign language (hello, goodbye, and yes); ∼8- to 18-month level during teen years	NR	Development reported as similar to that described by Baker et al.	Cognitive development appeared to be consistent with chronological age	Cognitive development reported as normal until 4 months “and then was retarded”	Currently uses three-word phrases with an assistive communication device and sign language
Feeding/nutrition	G-tube placed at 1.6 years; self-feeding at time of publication; g-tube use for medication and during periods of illness	“Fed a liquid mixture, augmented with trace elements”	NR	Some ability to self-feed; purees and thickened fluids	NR	Fed formula by mouth	NR	NR	G-tube placed at 2 weeks and removed at age 4; currently able to self-feed
Dermatological									
Acanthosis nigricans	Onset estimated to be at age 2	Onset at age 2	NR	Onset in teen years	Onset at age 13	Onset in “later life” of patient	NR	NR	Onset at age 2; underwent stages of surgical removal

^a^ Compared with Achondroplasia standards.

AED, antiepileptic drug; CNS, central nervous system; CSF, cerebral spinal fluid; FM, foramen magnum; MRI, magnetic resonance imaging; NR, not reported; SD, standard deviation; VP, ventriculoperitoneal.

#### Respiratory

Infants with TD face challenges with ventilation. We hypothesize that this is due to a combination of upper airway obstruction, tracheomalacia, abnormal pulmonary anatomy, and pulmonary hypoplasia due to small ribs and a narrow chest.^16^ Although we suspect that infants with TD who are unable to be ventilated despite all interventions are in the majority, we note that there is a subset of patients who have the potential to survive when offered respiratory support.

As in Charlie's case, all published individuals with TD who have survived beyond one year of life have required long-term mechanical ventilation (Table 1). Most patients required tracheostomy, although one described patient utilized long-term endotracheal intubation.^6,7^ Several individuals were able to experience brief ventilator-free windows throughout the day; however, there are no published reports before this one of individuals completely weaned off of invasive respiratory support. It is worth noting that one child has been reported to have tolerated periods of time off ventilation, but later developed neurological sequelae, leading to complete ventilator dependence by the end of her second decade.^9^

#### Neurological

As seen in achondroplasia, the anterior-posterior diameter of the foramen magnum can be significantly narrowed in TD. This may present with neurological changes, developmental delays, and/or central apnea. Foramen magnum decompression can provide relief of pressure on the spine for those with critical stenosis. We anecdotally witnessed significant improvements in all three of these domains for our patient after decompression surgery. However, as noted earlier, an individual has been described for whom there was progression of cervical spinal stenosis in the second decade of life despite decompression as a child, leading to quadriplegia as a teenager and regression to need of complete ventilator support.^9^

On brain imaging, a majority of children in the literature had ventriculomegaly and/or temporal lobe dysplasia or dysgenesis; however, only two children had a ventriculoperitoneal shunt placed (Table 1).^4,8,9,17–22^ Seizures were common, typically temporal lobe epilepsy, but seemed to be manageable with antiepileptic medications.^5,9^

Developmental delays are expected. The individual described in this study demonstrated slow and steady developmental progress over time, with intensive therapies and augmentative communication devices. Early intervention should be involved; however, physical therapy should avoid any manipulations of the neck or back so as to not exacerbate injury due to the condition-associated foramen magnum stenosis and thoracolumbar kyphosis. Expectations should not be that a child with TD will be conversant and ambulatory without assistive devices, but that there is potential for developmental advancement.^9^

#### Dermatological

Acanthosis nigricans is a consistent feature that appears to develop in individuals with TD. This is not a surprising finding as acanthosis nigricans is observed in several other *FGFR3*-related disorders.^23–25^ Using a lubricant in locations of friction can sometimes slow the progression and prevent skin breakdown. Alternatively, surgical excision can be beneficial for some children with widespread acanthosis if it is causing significant discomfort or risk of infection.

#### Growth

Growth velocity for children with TD is not known; however, the child in our case showed steady length, chest, and head circumference growth over time. We suggest growth points for a child with TD be best plotted on achondroplasia growth charts, with expectations that the head circumference would plot above the mean, and the height would plot more than two standard deviations below the mean. To this end, infants with TD inevitably have different weight goals, and we suggest a goal of 5 to 10 g/day in the first year of life. Weight gain above this amount can cause reflux, vomiting, and abdominal competition leading to increased respiratory needs.

### Potential for future treatment

C-type natriuretic peptide (CNP) is a potent positive regulator of endochondral bone growth. Studies have shown that CNP plasma levels are altered in *FGFR3*-opathies.^26^ Currently, clinical trials are underway testing the use of CNP analogues in children with achondroplasia. These studies are in progress, but early results show a modest increase in height.^27^ Although linear growth is one outcome being measured, the hope is that this treatment will decrease medical complications caused by skeletal changes in achondroplasia. There are also several other potential therapeutic agents for achondroplasia that are early in their development. We would be remiss if we did not mention the possibility of these medications being helpful to children with TD in the future as well. This is important to note as it further evidences the evolving clinical landscape for children with TD and informs the way in which palliative care physicians will need to counsel families about future potential and prognosis.

### The role of the palliative care team in an evolving landscape

The story of children such as Charlie surviving with a “lethal” disease is not unfamiliar in pediatric palliative care (PPC). Trisomy 13 and 18 (T13/18), once considered mostly fatal, are now well established as syndromes with wide phenotypic variation, and children are known to survive for decades both with and without surgical intervention.^28–30^ TD in some ways is today where T13/18 were in the 1980s—with single case reports of long-term survivors, such as Charlie, and parents fighting for medical providers to consider alternatives.^31^

Although we currently lack longitudinal data in TD, we can extrapolate that with surgical intervention (tracheostomy, gastric tube placement, foramen magnum decompression, and/or ventriculoperitoneal (VP) shunt when clinically indicated), survival curves are likely to differ from what we would expect with a “uniformly fatal” diagnosis. Quality of life (QOL), of course, is important to follow, along with survival. If Charlie is any indication, his parents perceive his QOL to be excellent and state they would do nothing differently if they were faced with the same medical decisions for Charlie all over again.

As with the paradigm shift that has occurred in the care of infants with T13/18, PPC teams have played and continue to play a pivotal role in advocating for goal-concordant clinical care that is based on best available evidence for the disease in question. With rare diseases such as TD, single case reports of survivors such as Charlie are important evidence that a condition may be life-limiting, but not necessarily “uniformly lethal.” We, therefore, recommend practitioners be wary of the label “lethal” and suggest counseling families on the variety of clinical outcomes documented in the literature.

## Abbreviations Used

AED: antiepileptic drug
CNS: central nervous system
CSF: cerebral spinal fluid
CNP: C-type natriuretic peptide
FM: foramen magnum
MRI: magnetic resonance imaging
NR: not reported
PPC: pediatric palliative care
QOL: quality of life
SD: standard deviation
TD: thanatophoric dysplasia
TD1: thanatophoric dysplasia type 1
T13/18: trisomy 13 and 18
VP: ventriculoperitoneal
